# SHC4 promotes tumor proliferation and metastasis by activating STAT3 signaling in hepatocellular carcinoma

**DOI:** 10.1186/s12935-022-02446-9

**Published:** 2022-01-15

**Authors:** Xin Zhang, Hongwei Zhang, Zhibin Liao, Jiacheng Zhang, Huifang Liang, Weixing Wang, Jia Yu, Keshuai Dong

**Affiliations:** 1 Department of General Surgery, Renmin Hospital of Wuhan University, Wuhan, China; 2 Hepatic Surgery Center, Tongji Hospital, Tongji Medical College, Huazhong University of Science and Technology, Wuhan, China; 3 Clinical Medical Research Center of Hepatic Surgery at Hubei Province, Wuhan, China; 4 Department of Hepatobiliary Surgery, Renmin Hospital of Wuhan University, Wuhan, China

**Keywords:** Hepatocellular carcinoma, SHC4, STAT3, Proliferation, Metastasis

## Abstract

**Background:**

The Src homology and collagen 4 (SHC4) is an important intracellular adaptor protein that has been shown to play a pro-cancer role in melanoma and glioma. However, the biological function and detailed mechanisms of SHC4 in hepatocellular carcinoma progression are unclear. This study aimed to evaluate the potential prognostic and treatment value of SHC4 in patients with HCC.

**Methods:**

The expression status of SHC4 in HCC tissues were investigated by immunohistochemistry and western blotting. Clinical significance of SHC4 was evaluated in a large cohort of HCC patients. The effects of SHC4 repression or overexpression on migration, invasion, and tumor growth were detected by colony formation assay, wound healing, transwell assays, and xenograft assay. Cell cycle and EMT-related proteins were detected by western blotting and immunofluorescence. In addition, the molecular regulation between SHC4 and STAT3 signaling in HCC were discovered by western blotting, immunofluorescence and xenograft assay.

**Results:**

SHC4 was overexpressed in HCC compared to adjacent normal liver tissues and increased SHC4 expression was associated with high AFP level, incomplete tumor encapsulation, poor tumor differentiation and poor prognosis. SHC4 was shown to enhance cell proliferation, colony formation, cells migration and invasion in vitro, and promotes cell cycle progression and EMT process in HCC cells. Tumor xenograft model assay confirmed the oncogenic role of SHC4 in tumorigenicity in nude mice. Moreover, activation of STAT3 signaling was found in the SHC4 overexpressed HCC cells and HCC tissues. Further intervention of STAT3 confirmed STAT3 as an important signaling pathway for the oncogenic role of SHC4 in HCC.

**Conclusions:**

Together, our results reveal that SHC4 activates STAT3 signaling to promote HCC progression, which may provide new clinical ideas for the treatment of HCC.

**Supplementary Information:**

The online version contains supplementary material available at 10.1186/s12935-022-02446-9.

## Introduction

Hepatocellular carcinoma (HCC) is one of the most common and highly malignant neoplastic diseases, with a global morbidity ranking sixth among all tumors and a mortality rate ranking third among tumor-related deaths [1]. HCC is a highly heterogeneous disease with multiple risk factors and etiology, including chronic hepatitis B or hepatitis C virus (HBV/HCV) infection, excessive alcohol consumption, aflatoxin exposure, and diabetes or obesity-related metabolic syndrome [2]. The underlying disease and difficulty in early diagnosis lead to poor prognosis and high mortality in HCC patients. Due to the serious lack of insight into the molecular machine of development and progression of hepatocellular carcinoma, we lack effective late-stage treatment options. Since sorafenib been approved as a systemic drug for HCC by FDA, lenvatinib, regorafenib, and cabozantinib have also been approved for the treatment of advanced HCC patients [3–5]. But it still shows unfavorable efficacy [6]. Therefore, it is urgent to further understand the mechanism of HCC progression so as to identify new drug targets for treatment.

The Src homology and collagen (SHC) family is one of the most studied adaptor protein families consisting of four members, SHC1, SHC2, SHC3, and SHC4. SHC1 is the most ubiquitous member of the family but limits to neurogenic areas in mature nervous system [7]. In contrast, SHC2 and SHC3 are expressed almost exclusively in neurons of the central and peripheral nervous systems [8]. SHC4 is also found in the adult brain, followed by skin and muscle [9–11]. It has been demonstrated that abnormal expression of SHC1 and SHC3 plays a role in malignant transformation, including transformation leading to HCC disease [12–15]. However, reports on SHC4 have been limited to studies of melanoma and glioma. High expression of SHC4 promoted the migration and invasion of melanoma cells and glioma cells [16, 17]. The function and regulatory mechanism of SHC4 in HCC are still unknown.

MAPK signaling pathways have been shown to play an important role in tumor progression that SHC1 and SHC3 mediated [18]. Involved in initiation of ERK pathway activation, JAK-STAT pathways intersects with MAPK pathways in multiple links. The activation of STAT requires MAPK auxiliary role as well. In HCC, aberrant activation of the JAK/STAT pathway promotes tumor growth, angiogenesis, invasion, and metastasis [19–21]. STAT3 is generally accepted as a bona fide oncogene in promoting HCC development. In our current study, we found increased SHC4 expression in HCC, leading to poor prognosis. We further confirmed the function of SHC4 in cell growth, migration and invasion. The significant role of STAT3 pathway in tumor progression is also involving, which may provide a new idea for the treatment of HCC.

## Materials and methods

### Clinical samples

The HCC tumor tissues and adjacent normal tissues of 138 patients were collected from resected specimens at Tongji Hospital of Huazhong University of Science and Technology between 2012 and 2016. Tissue microarray plates containing 105 HCC cases were constructed from paraffin-embedded HCC tissues. The diagnosis was based on the pathological examination. HCC staging was defined according to the criteria of the seventh edition of AJCC (American Joint Committee on Cancer) TNM classification. All patients received a standardized follow-up protocol, and the median follow-up was 25 months (range 0.5–65 months) [22]. Written informed consent for data analysis was obtained from all patients before operation.

### Plasmids and reagents and antibodies

SHC4 cDNA was kindly provided by Han’s Lab and cloned into pLenti-CMV-GFP vector to generate SHC4 expression plasmid which was confirmed by sequencing. The target sequences in the pLKO.1-SHC4 shRNA vector against human SHC4 were 5′-GCCTAGCATTTCTCAGTGTTT-3′, 5′-GAATGGCCCAAGACGTCATAA-3′ and 5′-ATGTTGCCTACGTAGCTAAAG-3′. pLenti-CMV-GFP and PLKO.1 were purchased from Addgene. Smartpool siRNA against human STAT3 was from RiboBio. The STAT3 siRNA target sequence was 5′-UCUACUUGGCUCCCAACUU dTdT-3′. Stattic was from MedChemExpress. Lipofectamine 2000 was from Invitrogen. Dulbecco’s modified Eagle’s medium (DMEM), Opti-MEM reduced serum media and fetal bovine serum (FBS) were from Gibco. Polybrene and puromycin were from Sigma. Primary antibodies used in this study include: SHC4 (Abcam, ab174908, 1:1000), GAPDH (Aksomics, KC-5G4, 1:10,000), c-Myc (Cell Signaling Technology, #5605, 1:1000), Cyclin D1 (Cell Signaling Technology, #2978, 1:1000), p21 (Cell Signaling Technology, #2947, 1:1000), E-cadherin (BD Biosciences, 610182, 1:1000), Occludin (Cell Signaling Technology, #5446, 1:1000), Slug (Cell Signaling Technology, #9585, 1:1000), STAT3 (Cell Signaling Technology, #12640, 1:1000) and p-STAT3 (Cell Signaling Technology, #52075, 1:1000).

### Cell culture, SHC4 overexpression and knockdown

HL7701, HL-7702, HepG2, Huh7, Sk-Hep1, Bel7402, HLE, HLF and Alex cell lines were purchased from China Center for Type Culture Collection (CCTCC, Wuhan, China). MHCC-97 H and HCC-LM3 cell lines were obtained from Liver Cancer Institute, Zhongshan Hospital, Fudan University, Shanghai, China. 293T cells were purchased from the American Type Culture Collection. Cells were cultured in DMEM (Hyclone, Logan, UT, USA) supplemented with 4.5 g/L glucose and 10% FBS (Gibco; Thermo Fisher Scientific, Inc.). All cell lines have been tested for their authenticity. Transfection were performed using Lipofectamine 2000 (Invitrogen; Thermo Fisher Scientific, Inc.) according to the manufacture’s instruction. For lentivirus production, pLKO.1 vector and pLKO.1-shSHC4 or pLenti-CMV-GFP vector and pLenti-CMV-GFP-SHC4 (6 µg), psPAX2 (4.5 µg), pMD2.G (1.5 µg) were purchased from Addgene, Inc. (Cambridge, MA, USA) and were co-transfected into 293T cells. The virus-containing supernatants were collected and filtered 48 h after transfection. Freshly made virus supernatants supplemented with 8 µg/mL polybrene (Sigma-Aldrich) were added to exponentially growing HepG2, Huh7 or HCC-LM3 cells. After 8 h, fresh medium was added. SHC4 stable overexpression or knockdown cells were achieved by 1-week puromycin (5 µg/mL, Ann Arbor, MI, USA) selection.

### Immunohistochemical staining

Immunohistochemistry (IHC) of clinical tumor samples and tumor xenograft samples was performed using antibodies against SHC4, Ki67 and E-cadherin. Briefly, tissue sections were deparaffinized in xylene, rehydrated with ethanol and subjected to antigen retrieval in boiling citrate buffer for 15 min. After peroxide block, the section was incubated with primary antibody at 4 °C overnight. The section was then treated with secondary antibody (Dako, Denmark) for 1 h at room temperature. The peroxidase reaction was developed with diaminobenzidine (DAB, Dako, Denmark). Images were acquired using 3DHIESTECH scan system and software. Cell-based average integrated option density (IOD) for SHC4 expression in each sample was analyzed by Image-Pro Plus 6.0 software (Media Cybernetics Inc, Bethesda, USA). The cutoff for the definition of high expression group or low expression group was the median value. Accordingly, samples were segregated into two groups for further analysis.

### Immunofluorescence staining

Cells were grown on coverslips in a 24-well culture plate, fixed with 4% paraformaldehyde for 15 min, permeabilized with 0.1% Triton X-100 for 15 min and blocked with 5% bovine serum albumin for 1 h. Cells were then incubated with primary antibodies overnight, followed by incubation with Alexa Fluor-conjugated secondary antibodies (Invitrogen, Carlsbad, CA) for 1 h. Finally, coverslips were incubated with DAPI (Sigma) for 5 min and visualized under an inverted fluorescent microscope.

### Western blotting

Cells or tissues were lysed in RIPA buffer supplemented with 1% protease (Roche) and 1% phosphatase inhibitor cocktail (Sigma). The protein samples were quantified using BCA assay, separated by sodium dodecyl sulfate-polyacrylamide gel electrophoresis (SDS-PAGE) and transferred onto polyvinylidene fluoride (PVDF) membranes (Millipore). After blocked with 5% skim milk, the membranes were probed with the indicated antibodies and then exposed to horseradish peroxidase (HRP)-linked secondary antibodies. The enhanced ECL was used for signal detection and western blot images were collected using Bio-Rad GelDoc system.

### Cell proliferation assay

HCC cells were seeded in 96-well microplate at the density of 1000 cells per well. After culture for 24, 48, 72, 96 and 120 h, the cells were treated with Cell Counting Kit-8 (CCK-8, Dojindo Laboratories) for 2 h at 37 °C. Then the optical density was measured at 450 nm using an enzyme-linked immunosorbent assay plate reader (Bio-Tek Elx 800, USA).

### Colony formation assay

HCC cells were seeded in 6-well plate at the density of 800 cells per wells. After 14 days, the colonies were fixed in 4% paraformaldehyde and stained with 1% crystal violet. The plates were taken picture and the number of colonies larger than 100 μm in diameter were counted.

### Transwell assay

Cell migration and invasion abilities were determined by Transwell assay as previous described [23]. In brief, cells in 200 µL of DMEM were placed in to the upper chamber of transwell inserts (8 μm, Corning, USA) for migration assay or Matrigel-coated transwell inserts for invasion assay, and 650 µL of 10% FBS-containing DMEM in the absence or presence of Stattic was added to the lower chamber. After incubation for 24 or 48 h, cells on the upper surface of the inserts were removed with a cotton swab. The migrative or invasive cells were fixed and stained. Photographs of six random fields across three replicate wells were captured for quantification analysis.

### Tumor xenograft model

BALB/c nude mice (4–6 weeks old, male) were purchased from HUAFUKANG (HUAFUKANG BIOSCIENCE CO. INC. Beijing, China). 2 × 10^6^ of Huh7 cells stably transfected with vector or SHC4 were subcutaneously inoculated into the flanks of nude mice. The mice were sacrificed by cervical dislocation 3 weeks after injection, and tumors were excised and weighed. Tumor volume was estimated according to the formula: volume = length × width^2^/2. The excised tumors were either embedded in paraffin for IHC analysis or snap frozen in liquid nitrogen for protein extraction. For the Stattic treatment, Huh7-vec and Huh7-SHC4 (2 × 10^6^/mouse) cells were subcutaneously implanted into the flanks of nude mice. When tumors grew to 3–5 mm in diameter, the mice were peritoneally treated with Stattic (50 mg/kg, three times per week for 4 weeks). Xenograft tumor samples were then collected for further analysis.

### Statistical analysis

Statistical analyses were performed using SPSS software (version 21.0, IBM Corp, Armonk, NY, USA) or the GraphPad Prism software (version 6.01, GraphPad Software Inc., San Diego, CA). Values were expressed as the mean ± SD from at least three independent experiments. Quantitative variables were compared using Student’s t-test or Mann–Whitney-U test when applicable. Also, the one-way ANOVA followed by a Turkey post hoc test was performed for multigroup comparison. The Pearson’s χ^2^ or Fisher’s exact test was used to analyze qualitative variables. Comparisons between Kaplan–Meier curves were performed using the log-rank test. *P *< 0.05 was considered statistically significant.

## Results

### SHC4 is significantly upregulated in HCC and positively associated with aggressive clinicopathological characteristics and poor prognosis in HCC patients

**Fig. 1:**
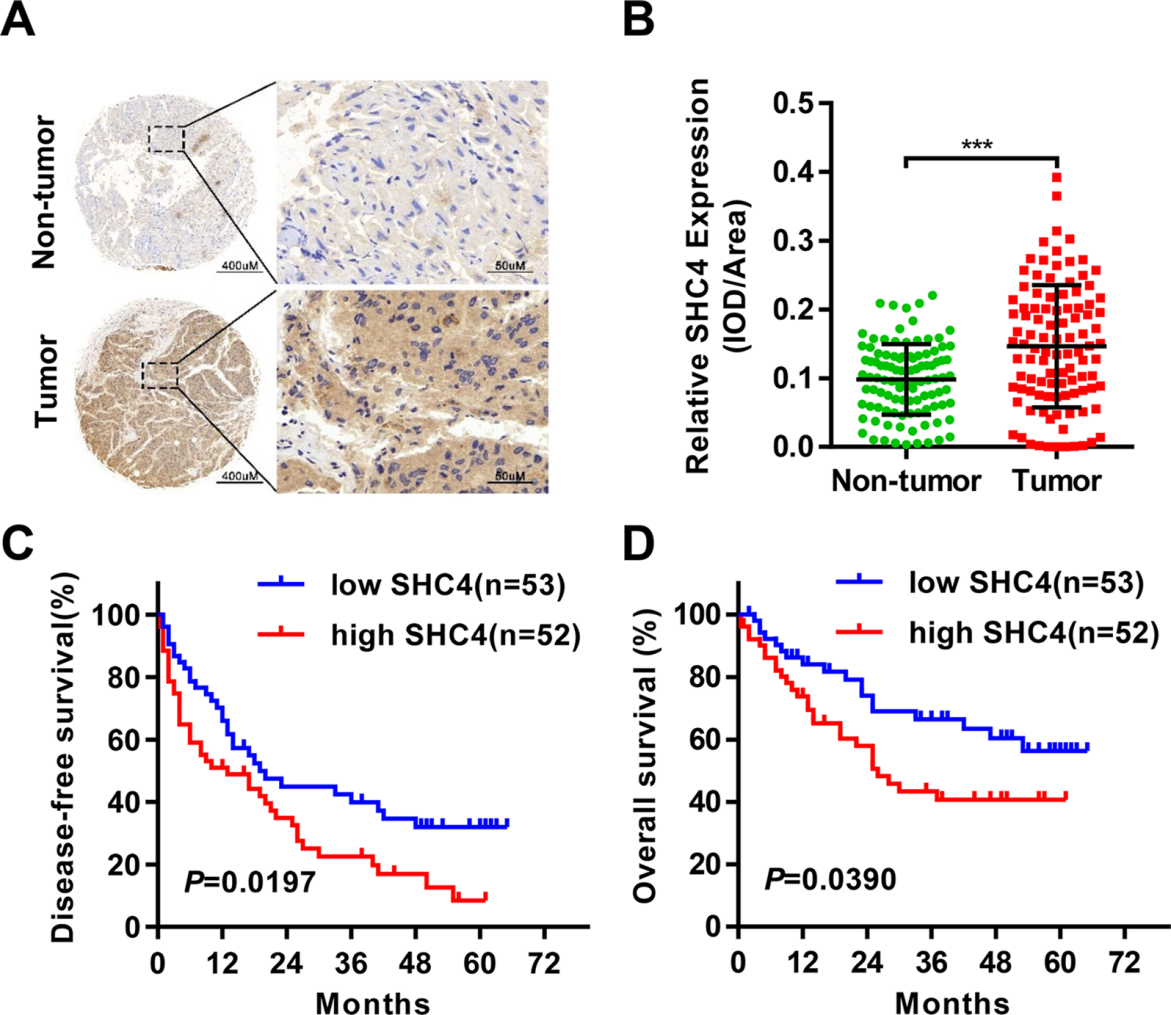
SHC4 expression is upregulated in HCC tissues and associated with poor prognosis of HCC patients. **A** Representative images of IHC staining for SHC4 in 105 paired HCC tumor and adjacent normal tissues. Scale bar, 400 μm (left panel) or 50 μm (right panel). **B** Dot density plot shows the distribution of average IOD for SHC4 level in the samples from **A**. ***P < 0.001, Paired t test. **C** Kaplan–Meier curves of disease-free survival rate of 105 HCC patients with SHC4 high or low expression levels. *P * = 0.0197, log-rank test. **D** Kaplan–Meier curves of overall survival rate of 105 HCC patients with SHC4 high or low expression levels. *P * = 0.0390, log-rank test

To investigate the relevance of SHC4 to HCC, we first examined SHC4 protein expression in a liver tumor tissue microarray containing 105 HCC tissue and adjacent normal liver tissues by IHC staining. We found that SHC4 was significantly overexpressed in HCC compared to adjacent normal liver tissues (Fig. 1A, B). To independently validate the findings, western blotting was carried out to examine the expression of SHC4 in paired tissues from additional 138 HCC patients. Consistent with the IHC analysis, SHC4 was highly expressed in most of the HCC samples (104/13,875.4%) (Additional file 1: Fig. S1A), and SHC4 was significantly higher in HCCs compared with their adjacent normal tissues (*P *< 0.0001, Additional file 1: Fig. S1B, C).

Next, we asked whether the expression of SHC4 was associated with clinicopathological characteristics and prognosis. According to IHC analysis, 105 patients were divided into two groups: low SHC4 group (n = 53) and high SHC4 group (n = 52). The detailed clinicopathological features is summarized in Table 1. Chi-squared analysis indicated that high SHC4 expression was notably associated with gender (*P * = 0.003), AFP level (*P * < 0.01), tumor encapsulation (*P * = 0.002), and tumor differentiation (*P  *= 0.023). TNM stage III–IV was more commonly found in patients with high SHC4 expression than those with low SHC4 expression, although significance was not demonstrated (*P  *= 0.167). Additionally, we found that female patients and patients with high AST and AFP, poor tumor differentiation, advanced TNM stage, incomplete tumor encapsulation appeared to exhibit high SHC4 expression (Additional file 1: Fig. S1D). Regarding the correlation of SHC4 expression with postoperative patient outcomes, Kaplan–Meier survival analysis revealed that patients with high SHC4 expression displayed significantly shorter disease-free survival (DFS) and overall survival (OS) periods than those with low expression (Fig. 1C, D). These results indicate that SHC4 is likely a carcinogenic factor in HCC.

**Table 1:** The correlation between SHC4 expression and clincopathological features in HCC

Clinical variables	No. of patients	SHC4 expression level		*P * value
	n = 105	Low (n = 53)	High (n = 52)	
Age (years)				0.668
< 60	83	41 (49.4%)	42 (50.6%)	
≥ 60	22	12 (54.5%)	10 (45.5%)	
Gender				0.003
Male	88	50 (56.8%)	38 (43.2%)	
Female	17	3 (17.6%)	14 (82.4%)	
HBsAg				0.381
Positive	81	39 (48.1%)	42 (51.9%)	
Negative	24	14 (58.3%)	10 (41.7%)	
ALT (U/L)				0.174
≤ 40	75	41 (54.7%)	34 (45.3%)	
> 40	30	12 (40.0%)	18 (60.0%)	
AST (U/L)				0.078
≤ 40	73	41 (56.2%)	32 (43.8%)	
> 40	32	12 (37.5%)	20 (62.5%)	
AFP (ng/mL)				< 0.001
< 400	57	38 (66.7%)	19 (33.3%)	
≥ 400	48	15 (31.3%)	33 (68.8%)	
Child–Pugh class				0.972
A	93	47 (50.5%)	46 (49.5%)	
B	12	6 (50.0%)	6 (50.0%)	
Liver cirrhosis				0.563
No	31	17 (54.8%)	14 (45.2%)	
Yes	74	36 (48.6%)	38 (51.4%)	
Tumor size(cm)				0.946
≤ 5	34	17 (50.0%)	17 (50.0%)	
> 5	71	36 (50.7%)	35 (49.3%)	
Tumor number				0.313
Single	83	44 (53.0%)	39 (47.0%)	
Multiple	22	9 (40.9%)	13 (59.1%)	
Tumor encapsulation				0.002
Yes	60	38 (63.3%)	22 (36.7%)	
No	45	15 (33.3%)	30 (66.7%)	
Vascular invasion				0.750
Yes	15	7 (46.7%)	8 (53.3%)	
No	90	46 (51.1%)	44 (48.9%)	
Tumor differentiation				0.023
Well	64	38 (59.4%)	26 (40.6%)	
Poor	41	15 (36.6%)	26 (63.4%)	
TNM stage				0.167
I–II	77	42 (54.5%)	35 (45.5%)	
III–IV	28	11 (39.3%)	17 (60.7%)	

### SHC4 promotes cell proliferation, colony formation and the expression of cell cycle proteins in HCC cells

**Fig. 2:**
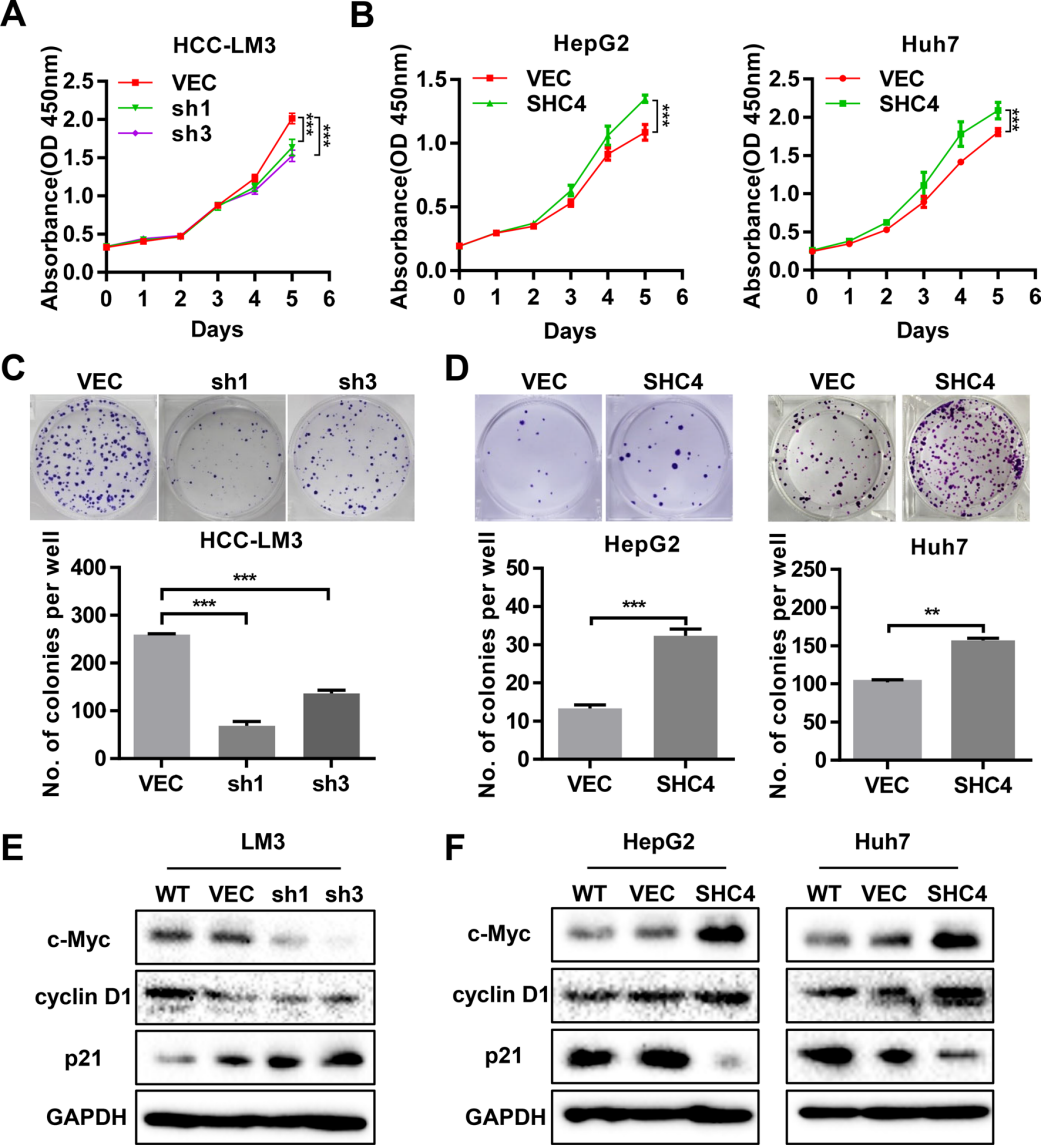
SHC4 promotes proliferation, anchorage-dependent colony formation, and regulates cell cycle genes in HCCs. **A**, **B** The cell viability of indicated stable cells was measured by CCK-8 assay at different time points. ***P  < 0.001, One-way ANOVA or Student t test. **C**, **D** The anchorage-depend colony formation assay was performed to assess the effects of SHC4 knockdown or overexpression on clone formation ability of HCC cells. Representative images of colonies were shown (upper panel) and the number of colonies were counted (lower panel). **P  < 0.01, ***P  <  0.001, One-way ANOVA or Student t test. **E**, **F** Effect of SHC4 knockdown or overexpression on cell cycle genes in indicated cells was identified by western blotting. Cell lysates were subjected to western blotting for c-MYC, cyclin D1, and p21

We performed immunofluorescence and immunohistochemistry staining to demonstrate that SHC4 localizes in the cytoplasm (Additional file 1: Fig. S2A, B). And western blotting was conducted to examine the protein expression of SHC4 in different liver and HCC cell lines (Additional file 1: Fig. S2C). Then, we chose HCC-LM3 cell as SHC4 high expression cell line and selected Huh7 and HepG2 cells as SHC4 low expression cell line for further experiments. HCC-LM3 cells were transduced with either 1 of 3 lentiviral shRNA targeting SHC4 or vector control. Western blotting was used to validate the reduction of SHC4 expression after shRNAs transduction (Additional file 1: Fig. S2D). According to the knockdown efficiency, we then selected shSHC4-1 and shSHC4-3 for a functional analysis. Conversely, stable ectopic expression of SHC4 in Huh7 and HepG2 cell lines were generated (Additional file 1: Fig. S2D). Cell proliferation assays demonstrated that knockdown of SHC4 in HCC-LM3 cell significantly inhibited cell viability compared with their vector controls (Fig. 2A), while an inverse effect was observed in Huh7 and HepG2 cells with SHC4 overexpression (Fig. 2B). In keep with this, knockdown of SHC4 decreased the number and size of colonies formation in HCC-LM3 cells (Fig. 2C), whereas ectopic expression of SHC4 enhanced the colony formation ability in Huh7 and HepG2 cells (Fig. 2D). Furthermore, cell cycle related proteins such as cyclin D1, c-Myc were attenuated and p21 was upregulated in SHC4 depleted HCC-LM3 cells (Fig. 2E). Concurrently, an inverse expression pattern of these markers was found in SHC4 overexpressed Huh7 and HepG2 cells, suggesting that SHC4 may boost cell growth by regulating cell cycle progression in HCC cells.

### SHC4 enhances cells migration and invasion abilities and promotes EMT of HCC cells in vitro

**Fig. 3:**
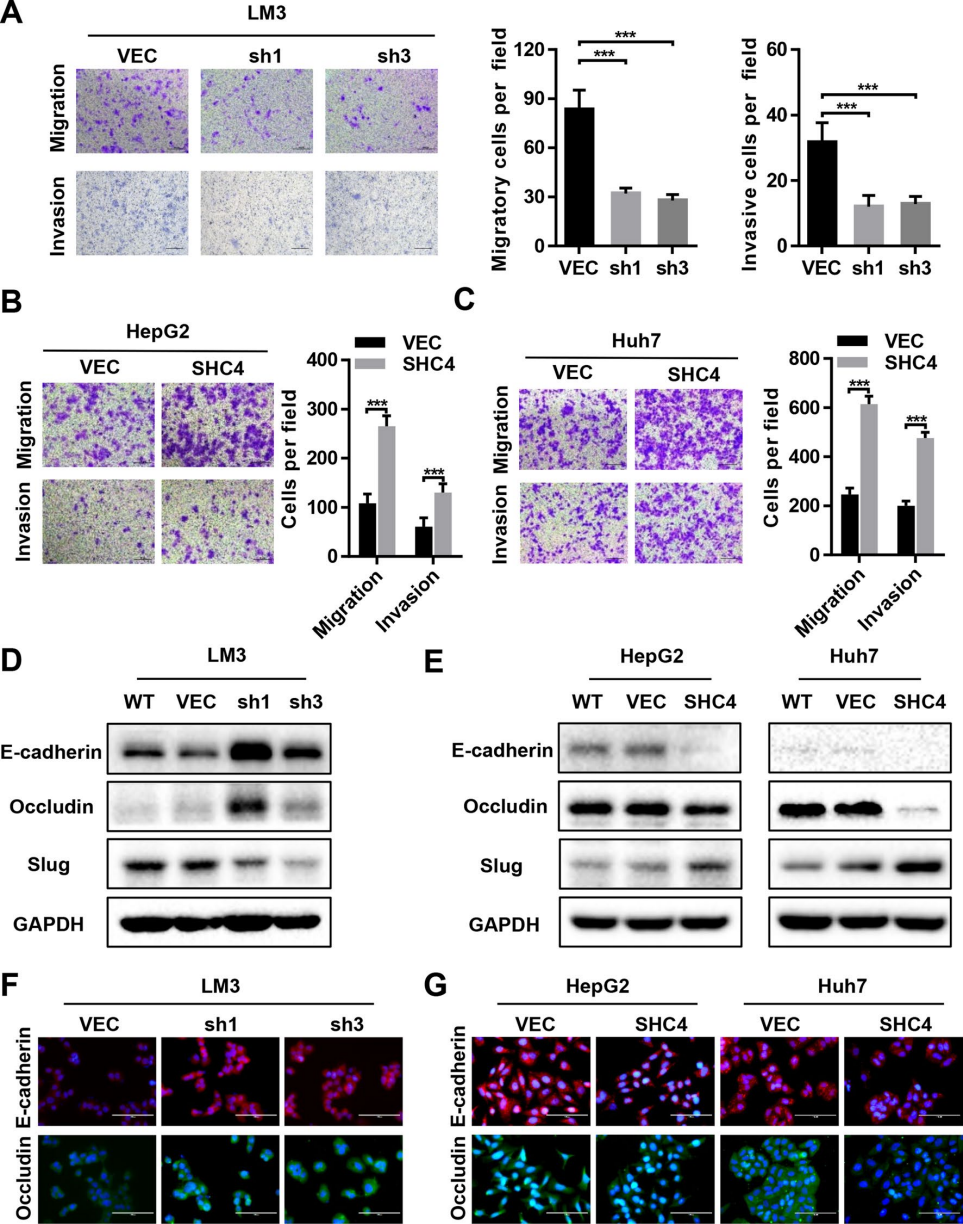
SHC4 positively regulated the migration, invasion and EMT of HCC cells. **A** Cells migration and invasion abilities in LM3 cells with SHC4 knockdown were determined by transwell assay. Representative image (left panel) and summary bar chart (left panel) are shown. Scale bar, 200 μm. ***P < 0.001, One-way ANOVA. **B**, **C** Cells migration and invasion abilities in HepG2 and Huh7 cells with SHC4 overexpression were determined by transwell assay. Representative image (left panel) and summary bar chart (left panel) are shown. Scale bar, 200 μm. ***P < 0.001, Student t test. **D**, **E** Expression of EMT markers, E-cadherin, Occludin and Slug in indicated cells were examined by western blotting. **F**, **G** Expression of EMT markers, E-cadherin and Occludin were assessed by immunofluorescence staining in indicated cells. Nuclei were visualized with DAPI (blue). Scale bars, 100 μm

To determine the effect of SHC4 on HCC cell metastasis, we conducted transwell assays to test the migration and invasiveness of these cells in vitro. Knockdown of SHC4 in HCC-LM3 cells attenuated the cells migrative and invasive capacities (Fig. 3A). In contrast, SHC4 overexpression in HepG2 and Huh7 cells enhanced their migrative and invasive abilities (Fig. 3B, C). EMT is adopted early during metastatic progression to permit invasion and migration of metastatic cells to secondary sites [24]. Hence, we examined EMT markers E-cadherin, Occludin and Slug by western blotting in above cells. The results indicated that epithelia markers, including E-cadherin and occludin were upregulated, but mesenchymal markers Slug was dramatically down-regulated in SHC4 knockdown HCC-LM3 cells (Fig. 3D). Furthermore, SHC4 overexpression induced the decrease of E-cadherin and Occludin expression and increase of Slug expression in both HepG2 and Huh7 cells (Fig. 3E). Similar results were also observed in immunofluorescence staining assays to detect these markers (Fig. 3F, G). Altogether, these results demonstrate that SHC4 is the crucial factor regulating cell migration and invasion, inducing EMT process in HCC cells.

### SHC4 induces the activation of STAT3 signaling

**Fig. 4:**
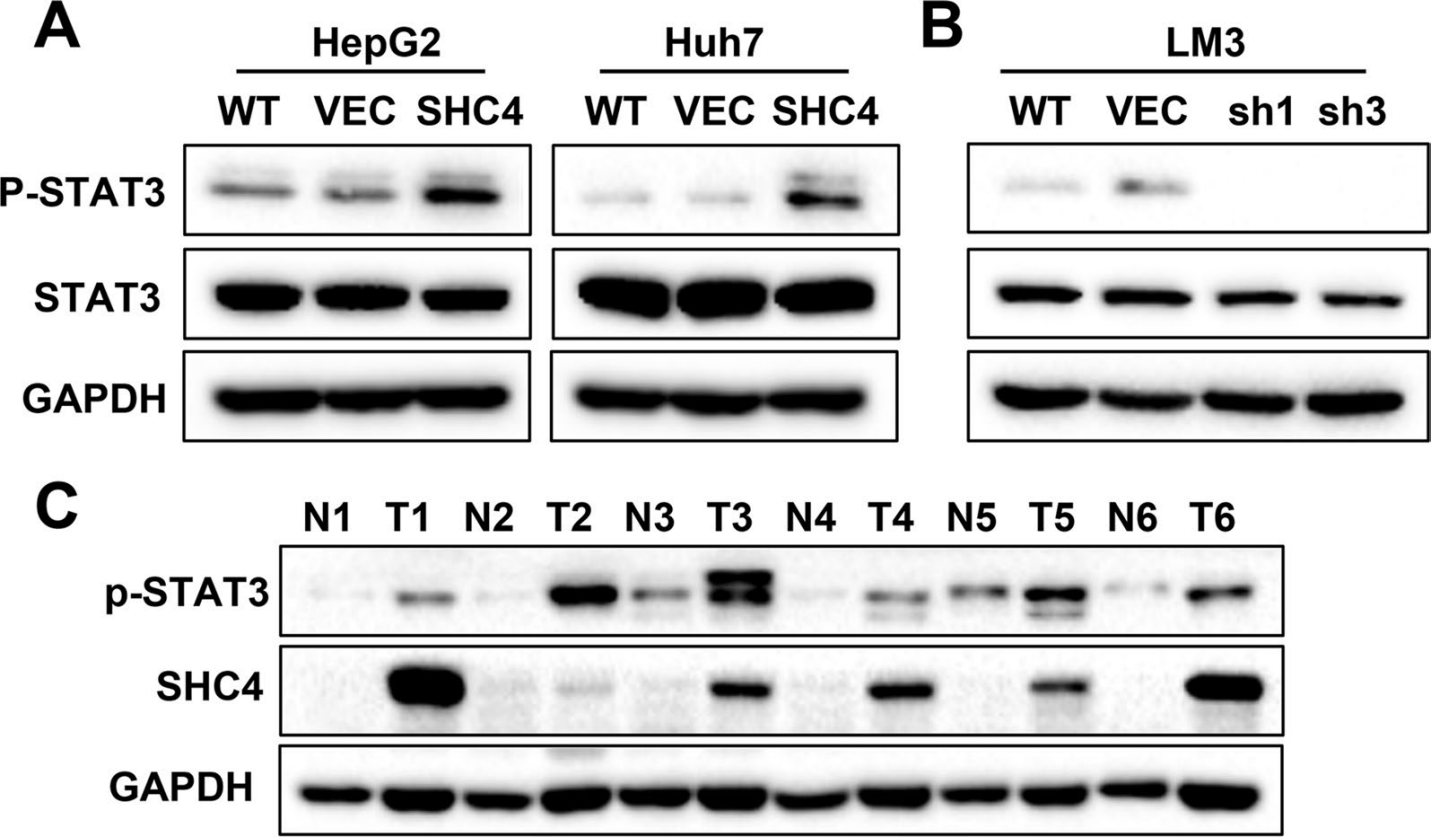
SHC4 promotes activation of STAT3 signaling pathway in HCC. **A** STAT3 and p-STAT3 levels were determine by western blotting in SHC4 overexpression cell lines. Stable overexpression of SHC4 enhanced the phosphorylation of STAT3 in HepG2 and Huh7 cells. **B** STAT3 and p-STAT3 levels were determine by western blotting in SHC4 knockdown cell line. Knockdown of SHC4 inhibited the phosphorylation of STAT3 in LM3 cells. **C** Protein expression of SHC4 and phosphorylation status of STAT3 in paired HCC tumor tissues were analyzed by western blotting

Then, we focused our minds on the downstream signaling pathways modulated by SHC4 in HCC. Several critical components in PI3K/AKT, JAK/STAT3, p53 and MAPK signaling pathways were screened, and the STAT3 signaling pathway had been evidently changed. As shown in Fig. 4A, SHC4 overexpression increased the phosphorylation of STAT3 in HepG2 and Huh7 cells but had no significant effect on the level of total STAT3. Moreover, the phosphorylation of STAT3 was lower in SHC4 knockdown HCC-LM3 cells compared to the control cells (Fig. 4B). We also detected the protein expression of SHC4 and phosphor-STAT3 in paired HCC tissues by western blotting. The results showed that the protein levels of SHC4 and phosphor-STAT3 were consistently higher in HCC tissues compared to their adjacent normal liver tissues (Fig. 4C). The above data suggest that SHC4 induces the activation of STAT3 signaling.

### The effect of SHC4 on cell proliferation, migration, invasion and EMT is dependent on STAT3 activation

**Fig. 5:**
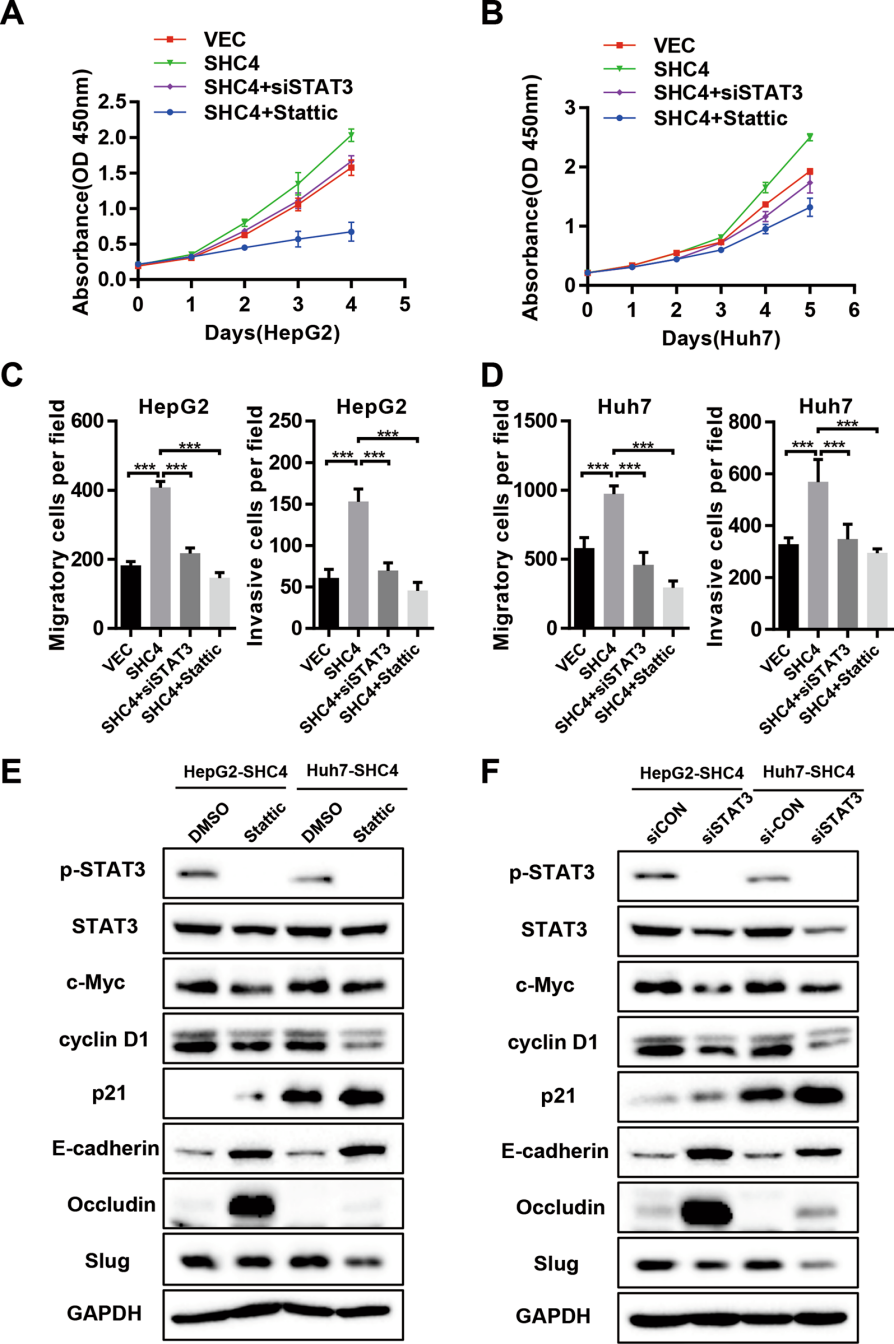
The oncogenic effect of SHC4 is relied on STAT3 activation. **A**, **B** HepG2 and Huh7 stably overexpressing SHC4 cells were transiently transfected siRNA targeting STAT3 or treated with Stattic (2 µmol/L). Cell viability of indicated cells were measured by CCK-8 assay. Knockdown of STAT3 or Stattic treatment reversed the increased proliferation induced by SHC4 overexpression in HepG2 and Huh7 cells. ***P < 0.001, Student t test. **C**, **D** Indicated cells were subjected to transwell assay to assess cell invasive and migrative abilities. Knockdown of STAT3 or Stattic exposure significantly attenuated the enhancement in cell migration and invasion induced by SHC4 overexpression. ***P < 0.001, Student t test. **E** Western blot was performed using specific antibodies for the p-STAT3, STAT3, c-Myc, cyclin D1, P21, E-cadherin, occluding and Slug in HepG2 and Huh7 stably overexpressing SHC4 cells with or without Stattic treatment. **F** HepG2 and Huh7 stably overexpressing SHC4 cells were transiently transfected siSTAT3 or siCON, then the expression of indicated markers were analyzed by western blotting

We next investigated whether STAT3 signaling is functionally crucial in SHC4-modulated cell proliferation, migration, invasion and EMT in HCC cells. HepG2 and Huh7 stably overexpressing SHC4 cells were transiently transfected siSTAT3 or treated with STAT3 inhibitor Stattic (2 µmol/L) for 48 h prior to cell proliferation and transwell assays. We found that cell growth was significantly suppressed following either siSTAT3 or Stattic treatment in SHC4 overexpressed HepG2 and Huh7 cells (Fig. 5A, B). Transwell assays indicated that siSTAT3 or Stattic exposure could remarkably reverse the effect of SHC4 on cell migration and invasion in both HeG2 (Fig. 5C and Additional file 1: Fig. S3A) and Huh7 cells (Fig. 5D and Additional file 1: Fig. S3A). Importantly, the results of western blotting analysis showed that siSTAT3 or Stattic efficiently abrogated the upregulation of c-Myc, cyclin D1 and Slug and rescued the decreased expression of E-cadherin, Occludin and p21 in SHC4 overexpressed HepG2 and Huh7 cells (Fig. 5E, F). Immunofluorescence analysis also suggested concomitant changes of EMT markers, such as E-cadherin and Occludin, when SHC4 overexpressed cells were exposed to siSTAT3 or Stattic (Additional file 1: Fig. S3B). Collectively, we conclude that SHC4 has an important role in controlling cell growth, migration, invasion and EMT by regulating the activation of STAT3 signaling.

### SHC4 promotes tumorigenicity in nude mice

**Fig. 6:**
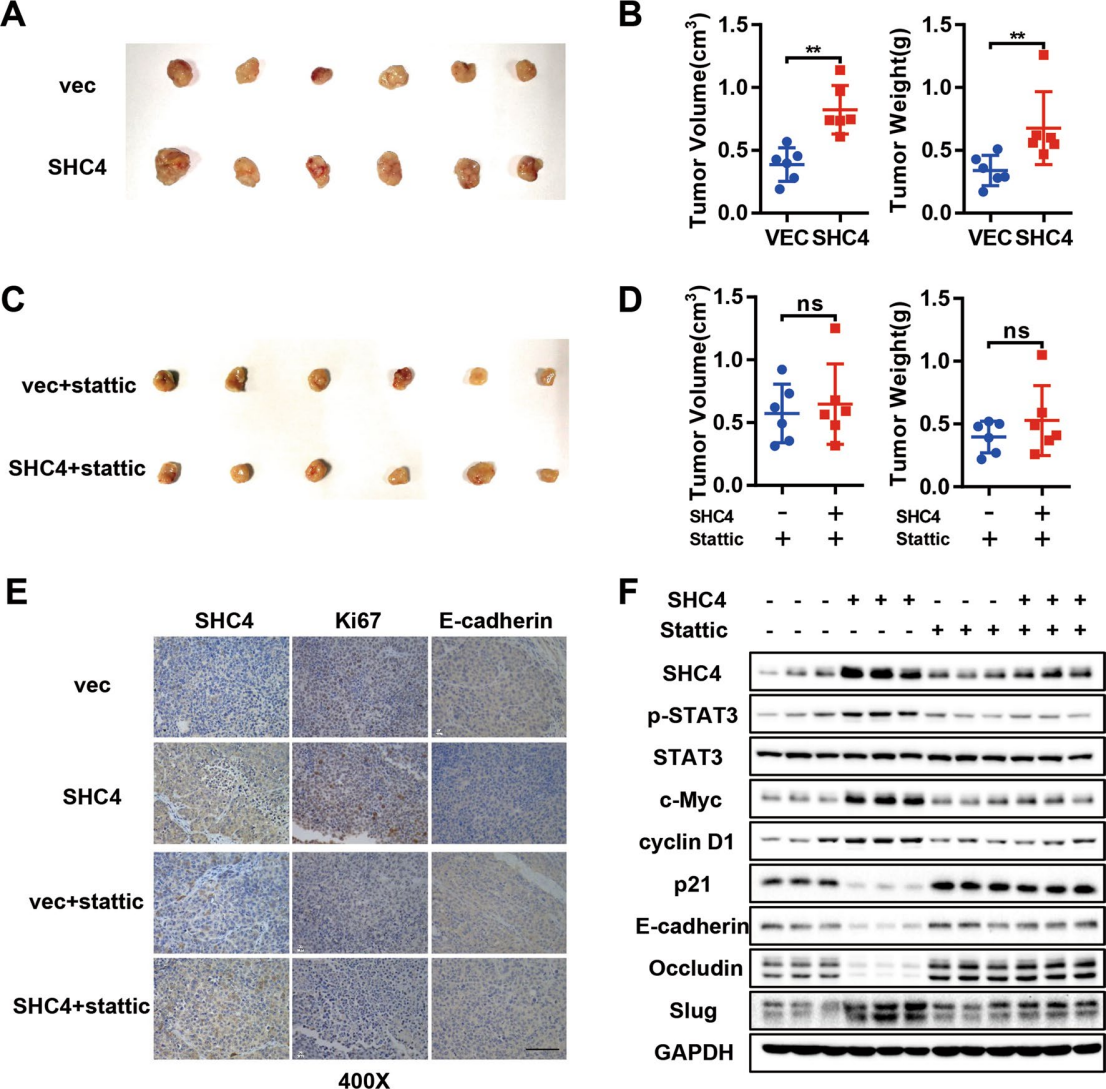
SHC4 overexpression enhances tumor growth in vivo, which could be blocked by Stattic. **A** Huh7 cells stably transfected with control or SHC4 were injected into the flank of nude mice. Photos for xenograft tumors isolated from nude mice 21 days post-inoculation. **B** Tumor volume and weight were compared between the two groups at the end of experiment. **P < 0.01, Mann–Whitney U test. **C** Huh7 cells stably transfected with control or SHC4 were injected into the flank of nude mice. After the tumors grew to 3–5 mm in diameter, mice were treated with Stattic (50 mg/kg, three times per week). Photos for xenograft tumors isolated from nude mice 30 days post-treatment. **D** Dot plots show the volume and weight of indicated tumors. *ns* no significance, Mann–Whitney U test. **E** The expression of SHC4, Ki67 and E-cadherin in indicated subcutaneous xenografts was determined by IHC. Scale bar, 50 μm. **F** The levels of SHC4, p-STAT3, STAT3, c-Myc, cyclin D1, p21, E-cadherin, Occludin and Slug were analyzed by western blotting from three representative xenograft tumors in each group

The oncogenic role of SHC4 was further investigated with an in vivo model. Huh7 stably overexpressing SHC4 or vector control cells were subcutaneously injected into the posterior flanks of nude mice. Tumor sizes and weights were measured 21 days after inoculation. SHC4 overexpressed Huh7 cells developed significantly larger tumors than vector control cells (Fig. 6A, B). To assess the therapeutic potential and examine the role of STAT3 in SHC4-induced tumorigenesis, we performed another subcutaneous xenograft model, in which nude mice were peritoneally treated with Stattic for 4 weeks after subcutaneous tumor reached 3–5 mm in diameter. Consistent with the function of STAT3 in vitro, inhibition of STAT3 by Stattic largely eliminated the enhancement in tumor growth induced by SHC4 overexpression (Fig. 6C, D). IHC staining confirmed the SHC4 overexpressed efficiency in xenograft tumor tissues (Fig. 6E). More proliferative cells were observed in SHC4 overexpressing xenografts as indicated by Ki-67 staining, but no obvious difference was observed between two groups with Stattic treatment (Fig. 6E). Similarly, the expression of E-cadherin was significantly suppressed in SHC4 overexpressed group, which was rescued by Stattic treatment (Fig. 6E). The expression levels of SHC4, p-STAT3, STAT3, c-Myc, cyclin D1, p21, E-cadherin, Occludin and Slug were also determined in xenograft tumors tissues by western blotting, which was in concordance with in vitro data (Fig. 6F). Taken together, these data indicate that SHC4 facilitates the tumorigenic ability of HCC cells through, at least in part, STAT3 signaling.

## Discussion

SHC4 was first identified and confirmed in melanoma in 2007. Ernesta Fagiani etc. found that SHC4 expression is essential for the growth of metastatic melanoma in vivo [17]. Subsequent studies indicated that SHC4 is highly expressed in malignant gliomas and it can promote invasion in U87 glioma cells [16]. SHC4 has also been reported to be expressed at high levels in adult brain tissue and at low levels in skeletal muscle [10, 11]. Melanie et al. detected enhanced SHC4 expression in astrocytomas and it was again shown to promote monolayer cell healing [25]. Furthermore, SHC4 widely expressed in the developing nervous system was further confirmed to be specifically expressed early during embryonic stem cell differentiation and embryonic development [9, 26]. On the basis of the studies above mentioned, the important role of SHC4 in the development of several tumors can be seen. In our current study, we for the first time showed the expression of SHC4 in HCC tissues and cell lines, with its high expression being associated with aggressive clinicopathological characteristics and poor prognosis in HCC patients. In consistent with the studies on glioma and melanoma, our functional analysis showed that SHC4 enhanced cancer proliferation and invasion abilities at both the cellular and organismal levels.

The typical structural domain of the SHC family is (CH2)-PTB-CH1-SH2. Although SHC articulators have a conserved structure and common binding partners, the SHC adaptors and their isoforms can generate distinct downstream signals and make unique physiological contributions [27]. The molecular mechanisms of SHC1-mediated signal transduction have been widely documented: SHC1 bind to the cell membrane receptor through PTB domain, promoting CH1 domain binding to Grb2 and solicits SOS to form SHC1-Grb2-SOS complex, and further activates Raf/MEK/MAPK signaling pathway [13]. Along this context, Yun Liu and others found that SHC3 forms a complex with MVP, MEK, and ERK, which potentiates ERK activation, independent of the classic SHC1, Grb2, SOS, Ras, and Raf pathway [15]. However, the mechanism of SHC4 action in cancer cells, especially in HCC, largely remains unknown.

SHC4 promotes migration and invasion of melanoma cells through activation of Ras-dependent or Ras-independent pathways [17, 28]. SHC proteins are involving in the epidermal growth factor receptor (EGFR) internalization process and the increased expression level of SHC4 in glioma promoted the phosphorylation of EGFR specific sites [16, 25, 29]. Recent studies have shown that SHC4 regulate EV secretion and promote tumorigenesis of prostate [30]. During the process that embryonic stem cells differentiate into ectodermal stem cells, SHC4 knockout promoted the enrichment of CDX2-positive cells and result in the activation of MAPK-ERK1/2 [26]. In our study, we explored the potential mechanisms of SHC4 in HCC by analyzing the transcription factor STAT3. Belongs to the signal transducer and activator of transcription (STAT) family, STAT3 is inactive in non-stimulated cells but is rapidly activated by various cytokines and growth factors, for instance, the receptor-associated Janus kinase (JAK) [31, 32]. Activated STAT3 plays a role in most cancers, mediating the expression of various genes in response to cellular stimulation and playing an important role in cell growth and apoptosis [33]. Notably, STAT3 has been widely recognized to play an important role in the progression and treatment of HCC [34–37]. Nonetheless, the exact mechanism of STAT3 activation in HCC remains unclear. In this study, we confirmed that SHC4 overexpression can activate STAT3. Further research on cell lines showed that knockdown and inhibition of STAT3 attenuates the proliferation, migration and invasion of tumor cells caused by overexpression of SHC4. Experiments on mice had also been conducted to verify that SHC4 promoted tumor growth, and Stattic could reverse this effect. Therefore, STAT3 was essential for the effects of SHC4 on HCC cell proliferation, migration and invasion. STAT3 inhibitors may be a potential option for liver cancer treatment.

As is mentioned above, studies on how SHC1 and SHC3 activate downstream molecules are well established. Even though we have proposed a new viewpoint on HCC progression, deeper mechanisms how SHC4 activate STAT3 signaling is yet to be explored. Acting as an adaptor protein, SHC4 itself don’t have the activity of protein kinase. Therefore, it usually interacts with other proteins to regulate signal transduction, so our further study will focus on the molecular mechanisms underlying the SHC4 induced STAT3 signaling activation.

## Conclusions

In conclusion, overexpression of SHC4 potentiates STAT3 activation and stimulates a repertoire of downstream tumorigenic responses, including proliferation, migration, invasion, and EMT, leading to aggressive clinicopathological characteristics and poor prognosis in HCC patients. These evidences support the feasibility of SHC4 as novel therapeutic target for HCC.

## Supplementary Information

**Additional file 1: Figure S1.** SHC4 is overexpressed in HCC tissues and associated with aggressive clinicopathological characteristics. **Figure S2.** SHC4 knockdown or overexpression in HCC cell lines. **Figure S3.** STAT3 inhibition blocks the promotive effect of SHC4 on migration, invasion and EMT in HCC cells.

## Data Availability

All data generated or analyzed during this study are included in this published article.

## Abbreviations

AST: Aspartate aminotransferase
ALT: Alanine aminotransferase
AFP: Alpha-fetoprotein
SHC: The Src homology and collagen
HCC: Hepatocellular carcinoma
EMT: Epithelial–mesenchymal transition
FDA: Food and Drug Administration
AJCC: American Joint Committee on Cancer
shRNA: Short hairpin RNA
siRNA: Small interfering RNA
OS: Overall survival
DFS: Disease-free survival
AJCC: American Joint Committee on Cancer
IOD: Integrated option density
IHC: Immunohistochemistry
