# Supplementary material for: SHC4 promotes tumor proliferation and metastasis by activating STAT3 signaling in hepatocellular carcinoma
Source: Cancer Cell Int. 2022 Jan 15;22:24. doi: 10.1186/s12935-022-02446-9 (PMC8760801; doi:10.1186/s12935-022-02446-9)
Supplement: Supplementary file 1 — Additional file 1: Figure S1. SHC4 is overexpressed in HCC tissues and associated with aggressive clinicopathological characteristics. Figure S2. SHC4 knockdown or overexpression in HCC cell lines. Figure S3. STAT3 inhibition blocks the promotive effect of SHC4 on migration, invasion and EMT in HCC cells. [file 12935_2022_2446_MOESM1_ESM.docx]

**Additional file 1**


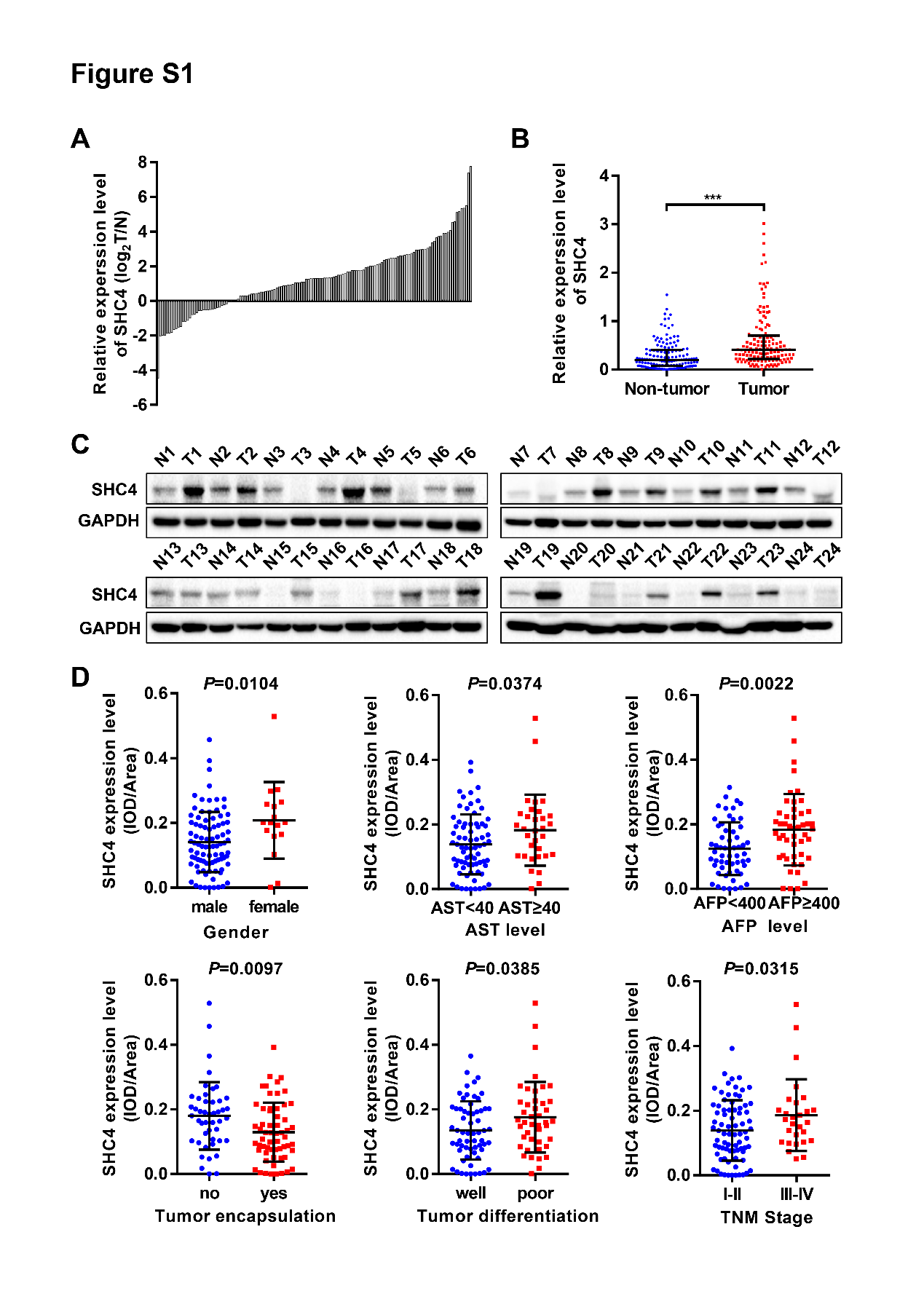


**Fig. S1 SHC4 is overexpressed in HCC tissues and associated with aggressive clinicopathological characteristics.** (A) SHC4 protein expression in 138 paired HCC tumor and adjacent normal tissues were detected by western blotting. SHC4 level of HCC tissues were quantified in the bar chart after normalized to their paired adjacent normal tissues. (B) Difference in SHC4 levels between HCC tissues and their paired adjacent normal tissues was quantified. The expression of SHC4 was normalized against GAPDH, according to the intensity of each lane with a computerized image system (Image Lab version 5.2.1, Bio-Rad laboratories). ***, *P*<0001, Paired t test. (C) Representative images of western blotting in 24 paired HCC tumor and adjacent normal tissues are shown. T, tumors; N, normal tissues. (D) Dot density plots depict relative SHC4 expression identified by average IOD in different groups of HCC tissues, male versus female (*P*=0.0104), AST< 40 U/L versus AST ≥ 40 U/L (*P*=0.0374), AFP < 400 ng/ml versus AFP ≥ 400 ng/ml (*P*=0.0022), with tumor encapsulation versus without tumor encapsulation (*P*=0.0097), well tumor differentiation versus poor tumor differentiation (*P*=0.0385), and TNM stage Ⅰ-Ⅱ versus stage Ⅲ-Ⅳ (*P*=0.0315). Student t test.


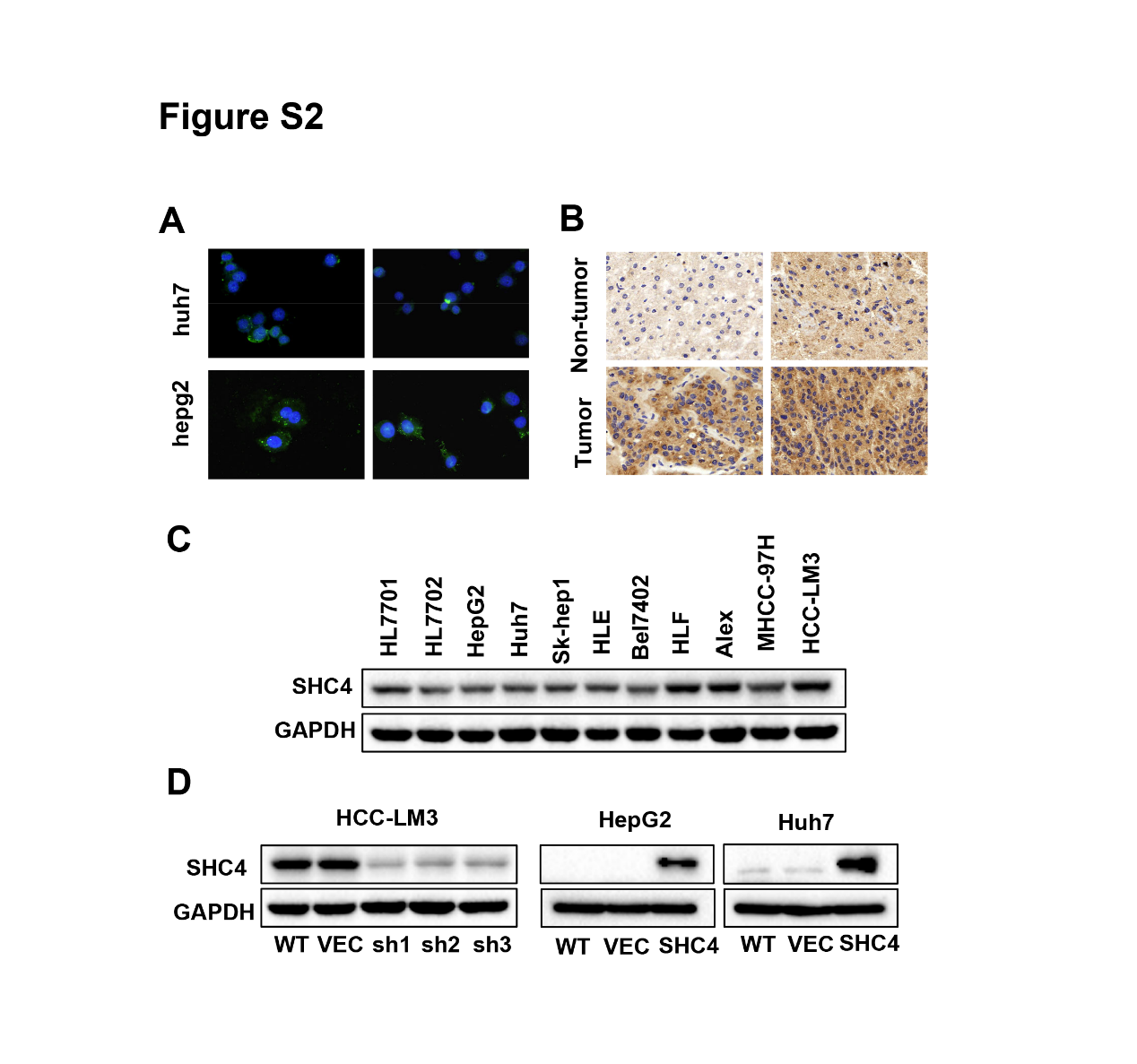


**Fig. S2. SHC4 knockdown or overexpression in HCC cell lines.** (A) Expression of SHC4 was assessed by immunofluorescence staining in indicated cells. Nuclei were visualized with DAPI (blue). (B) Representative images of immunohistochemistry staining for SHC4 in 2 paired HCC tumor and adjacent normal tissues. (C) Relative SHC4 expression levels in different liver and HCC cell lines were analyzed by western blotting. GAPDH was used as a loading control. (D) Western blot analysis confirmed the knockdown efficiency of SHC4 by shSHC4 in HCC-LM3 cell line and ectopic expression of SHC4 in HepG2 and Huh7 cell lines.


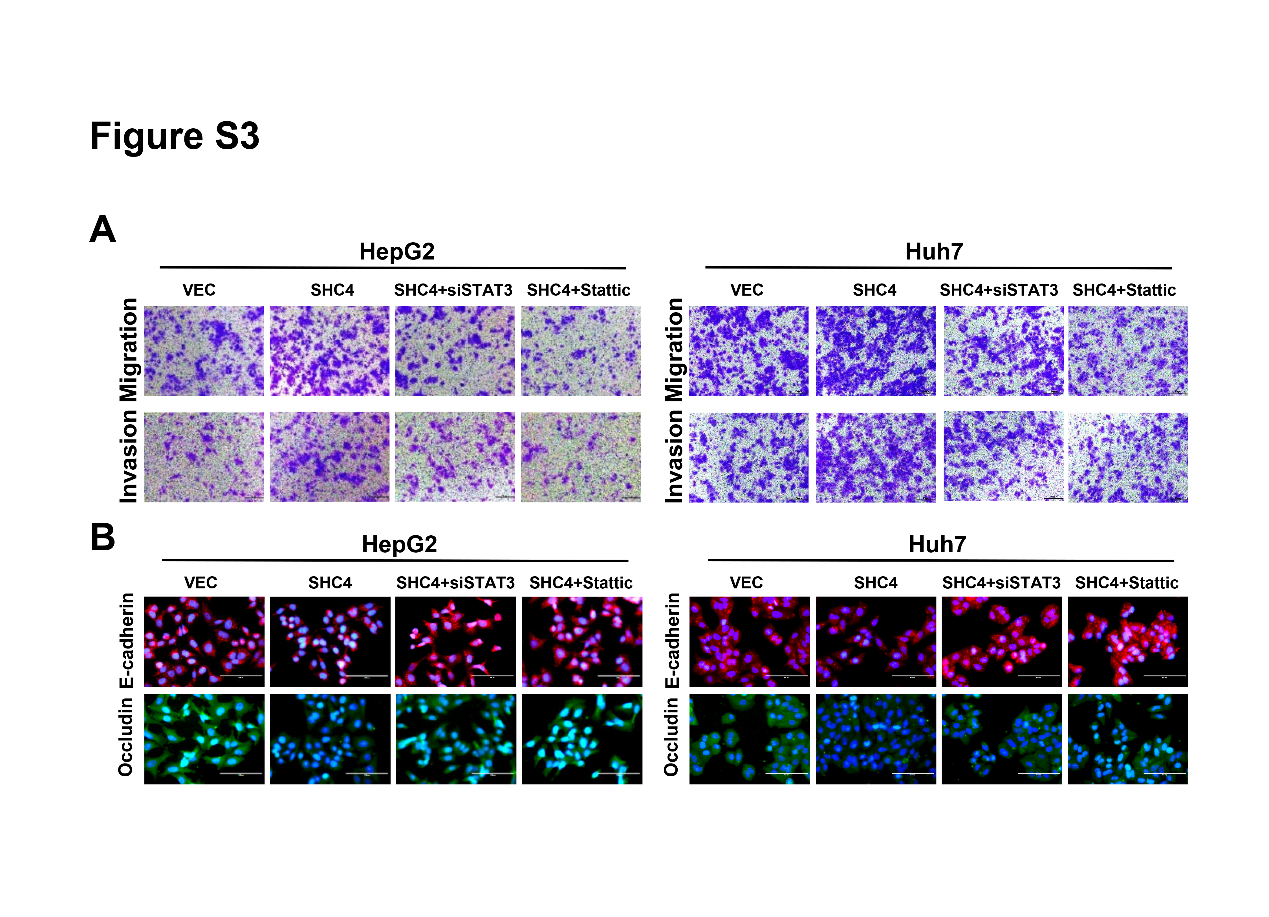


**Fig. S3 STAT3 inhibition blocks the promotive effect of SHC4 on migration, invasion and EMT in HCC cells.** (A) Cell invasion and migration were estimated in HepG2 and Huh7 cells overexpressed with SHC4 alone or co-treated with siSTAT3 or Stattic. Representative images are shown. Scale bar, 200 μm. (B) Representative immunofluorescence images of E-cadherin and Occludin in HepG2 and Huh7 cells overexpressed with SHC4 alone or co-treated with siSTAT3 or Stattic. Nuclei were visualized with DAPI (blue). Scale bars, 100 μm.
